# Waiting-time statistics in magnetic systems

**DOI:** 10.1038/s41598-020-66727-x

**Published:** 2020-06-16

**Authors:** Ivandson Praeiro de Sousa, Gustavo Zampier dos Santos Lima, Marcio Assolin Correa, Rubem Luis Sommer, Gilberto Corso, Felipe Bohn

**Affiliations:** 1 Departamento de Física, Universidade Federal do Rio Grande do Norte, 59078-970 Natal, RN Brazil; 2 Escola de Ciências Tecnologia, Universidade Federal do Rio Grande do Norte, 59078-970 Natal, RN Brazil; 3 Departamento de Biofísica e Farmacologia, Universidade Federal do Rio Grande do Norte, 59078-970 Natal, RN Brazil; 4 Centro Brasileiro de Pesquisas Físicas, Rua Dr. Xavier Sigaud 150, Urca, 22290-180 Rio de Janeiro, RJ Brazil

**Keywords:** Condensed-matter physics, Statistical physics, thermodynamics and nonlinear dynamics

## Abstract

Many complex systems, from earthquakes and financial markets to Barkhausen effect in ferromagnetic materials, respond with a noise consisting of discrete avalanche-like events with broad range of sizes and durations, separated by waiting times. Here we focus on the waiting-time statistics in magnetic systems. By investigating the Barkhausen noise in amorphous and polycrystalline ferromagnetic films having different thicknesses, we uncover the form of the waiting-time distribution in time series recorded from the irregular and irreversible motion of magnetic domain walls. Further, we address the question of if the waiting-time distribution evolves with the threshold level, as well as with the film thickness and structural character of the materials. Our results, besides informing on the temporal avalanche correlations, disclose the waiting-time statistics in magnetic systems also bring fingerprints of the universality classes of Barkhausen avalanches and a dimensional crossover in the domain wall dynamics.

## Introduction

Many physical systems in nature crackle^1^. An example of crackling may be as prosaic as a piece of paper that exhibits intermittent sharp noise when it is slowly crumpled^2^. Or, on a macro-scale, the Earth’s surface also responds with violent and intermittent earthquakes as the tectonic plates rub past one another^3^. Further, remarkably, crackling has been yet detected in the avalanche behavior of for instance micro-fracturing phenomena^4^, in the dynamics of superconducting vortices^5^, in the Barkhausen effect in ferromagnetic materials^6–9^, and even in the financial markets^10^. Despite crackling is found in fundamentally different systems, such systems often share general features. Which means, when slowly driven, these systems respond with a noise consisting of discrete avalanche-like events with broad range of sizes and durations, separated by waiting times; and the emitted noise across all of them is characterized by avalanches with scale-invariant properties, power-law distributions, and universal features^11^.

Much efforts have been devoted to characterize the dynamics of crackling systems, thus providing insights on the nature into these critical phenomena. Within this field, statistical mechanics of unbalanced systems with phase transitions has inspired numerous investigations in recent decades^12^. In addition, stochastic theories about turbulent processes^13^ and complex systems have motivated new approaches to the understanding of the crackling noise^14–16^. Last but not least, it shall be also highlighted the advances of the theory of self-organized systems, a fruitful area of out-of-equilibrium thermodynamics^17,18^.

Generally, the task of analyzing the critical dynamics in complex systems is essentially done by scrutinizing the statistical properties of the avalanches in global activity time series^19^. To this end, by imposing an arbitrary threshold, one can distinguish notably two parts in the crackling noise time series: the first part consists in the avalanches themselves, which are characterized by large amplitudes in the signal, above the threshold; the second one in turn consists in the low-amplitude noise, below the threshold, thus corresponding to the time interval between two successive avalanches, i.e. the waiting time.

Numerous statistical functions have been proposed to evaluate the avalanche statistics. These are the cases of for instance the distributions of avalanche sizes and avalanche durations, the average avalanche size as a function of its duration, the power spectrum, and the average temporal avalanche shape. Focusing just on the temporal avalanche statistics, it is well known that the distribution of avalanche durations follows a cutoff-limited power-law scaling behavior, which is associated to a scaling exponent^9^. This exponent is in general independent of the threshold level, thus reflecting general features of the underlying system dynamics and being a signature of an universality class. However, while the avalanche durations have been widely investigated, the same effort was not intended to the analysis of the waiting time. As a consequence, many questions on the statistics of the time interval between the avalanches still remain elusive. Among them, perhaps the most remarkable doubts on the issue resides in the form of the waiting-time distribution, with its implications regarding the temporal avalanche correlations, and the effects of the thresholding process on its analysis.

In this letter, we look at the waiting-time statistics in magnetic systems by investigating the Barkhausen noise in amorphous and polycrystalline ferromagnetic films having different thicknesses. Specifically we ask whether the waiting-time distribution in time series recorded from the irregular and irreversible motion of magnetic domain walls (DW) in magnetic systems is characterized by power-law and/or exponential laws. Further, we address the question of if the waiting-time distribution evolves with the threshold level, as well as with the film thickness and structural character of the materials.

## Results

### Waiting-time statistics in magnetic systems

**Figure 1:**
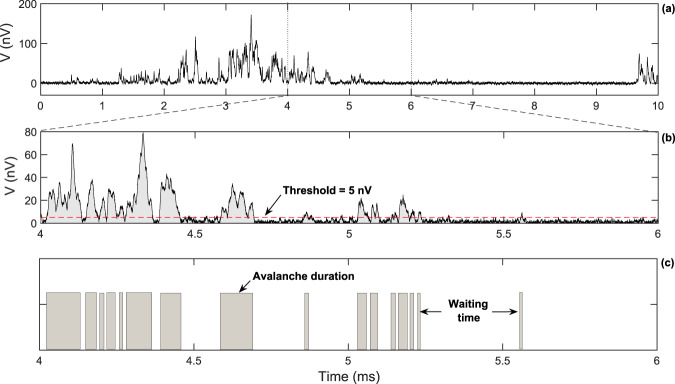
Defining waiting times in magnetic noise with the threholding process. (**a**) Representative example of the experimental Barkhausen noise time series measured in ferromagnetic films. (**b**) A zoom over a smaller time scale evidences the voltage pulses combined with background instrumental noise, as well as self-similarity properties. By imposing a finite threshold level (red dashed line, in this case of 5 nV), we define the avalanches, with their sizes and durations, and the waiting times. (**c**) When the signal has excursions below the threshold, the corresponding time intervals between the avalanches (gray rectangles) are referred to as the waiting times (blank spaces).

Barkhausen noise is a fingerprint of the complex microscopic magnetization process through the jerky motion of magnetic domain walls in ferromagnetic materials^6–9^. In the presence of a smooth, slow-varying external magnetic field, the material responds through a sequence of discrete and irregular jumps of magnetization which can be detected as a crackling noise by a pickup coil wound around the ferromagnetic material. In a typical Barkhausen noise experiment, as the magnetization changes, the respective variation of the magnetic flux induces a voltage signal (as we can see in Fig. 1a) in the coil that can be amplified and recorded. Here we focus on the waiting-time statistics in Barkhausen noise time series recorded in amorphous FeSiB and polycrystalline NiFe ferromagnetic films with thicknesses from 50 to 1000 nm. Thereby, besides checking the form of the distribution, we probe for the impacts of the threshold level (Fig. 1b) and of the film thickness and structural character of the materials on the waiting-time statistics (Fig. 1c) in magnetic systems (see Methods for details on the films, experiment and statistics of the noise).

### The exponential-like distribution of waiting times

**Figure 2:**
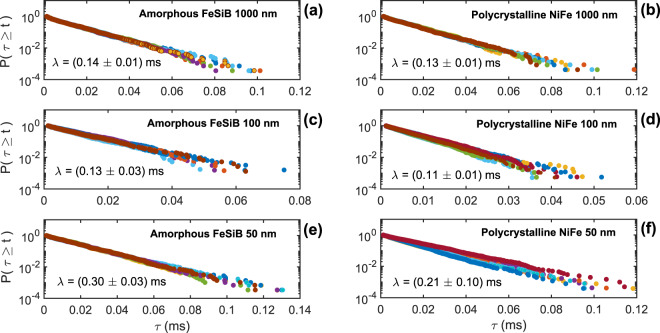
The exponential-like distribution of waiting times. Cumulative distribution function of waiting times $P(\tau \ge t)$ measured from Barkhausen noise time series recorded in amorphous FeSiB and polycrystalline NiFe ferromagnetic films having thicknesses of 1000, 100, and 50 nm. In particular, here we present 50 representative waiting-time distributions obtained imposing a threshold level of 5 nV, selected from the set of 200 time series analyzed for each sample. Remarkably, the cumulative distribution function of waiting times $P(\tau \ge t)$ follows a robust exponential behavior, which can be fitted by $P(\tau \ge t)\propto {e}^{-\tau /\lambda }$, thus providing *λ*, the characteristic time of the exponential process.

Figure 2 illustrates the waiting-time statistics for amorphous and polycrystalline films having different thicknesses. In particular, we plot here the cumulative distribution function of waiting times $P(\tau \ge t)$ in order to improve the accuracy of the waiting-time statistics, given that fluctuations are weakened in the cumulative sum (see Methods for details on the analysis).

Despite the intense debate in literaure in last years^19–23^, theoretical predictions, numerical calculations and experiments led controversial interpretations, raising doubts on the precise form of the distribution of waiting times. However, from our results in magnetic systems, a closer examination of $P(\tau \ge t)$ in a log-linear plot clearly reveals a robust exponential behavior. Remarkably, all samples behave in a similar manner, irrespective of the film thickness and the structural character of the materials. Thereby, the cumulative distribution function of waiting times may be well fitted considering $P(\tau \ge t)\propto {e}^{-\tau /\lambda }$, which depends on a single parameter *λ*, the characteristic time of the exponential process. Specifically, the exponential law uncovers the existence of characteristic scale, i.e. a typical inter-avalanche time in the Barkhausen signal.

### The effect of threshold level on temporal avalanche statistics

**Figure 3:**
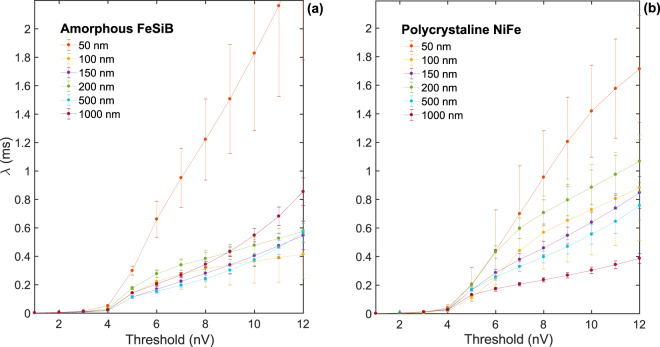
The effect of threshold level on the temporal avalanche statistics. Characteristic time *λ* of the exponential process, i.e. typical inter-avalanche time, estimated from the exponential-like distributions of waiting times measured from Barkhausen noise time series recorded in amorphous FeSiB and polycrystalline NiFe ferromagnetic films with different thicknesses. The distributions of waiting times were obtained imposing distinct threshold levels in order to verify the impacts of our choice on the statistical results. The error bars were estimated using the standard deviation.

Figure 3 brings the evolution with the threshold of the typical inter-avalanche time *λ* in the Barkhausen signal for amorphous and polycrystalline films having different thicknesses.

For threshold values below 5 nV, we observed *λ*~0 ms, a fact due to the divergence of the waiting-time distributions for very small $\tau$ values. This result is a direct consequence of the presence of an experimental background noise, with amplitude of ~4 nV, which introduces white-noise-like features to the signal that dominate the time series behavior. At this condition, we understand that the waiting-time statistics become meaningless, not bringing information of the magnetic process associated to the Barkhausen noise. Therefore, from now on we focus our attention on the results obtained for threshold values chosen above the background noise. In this context, we find *λ* values within the range between 0.1 and 2.0 ms. As expected, these values are modified with the threshold level. However, it is interesting to notice that, as a general trend, *λ* continuously raises with the increase of the threshold.

### Dependence of the waiting-time statistics with film thickness and structural character of the materials

**Figure 4:**
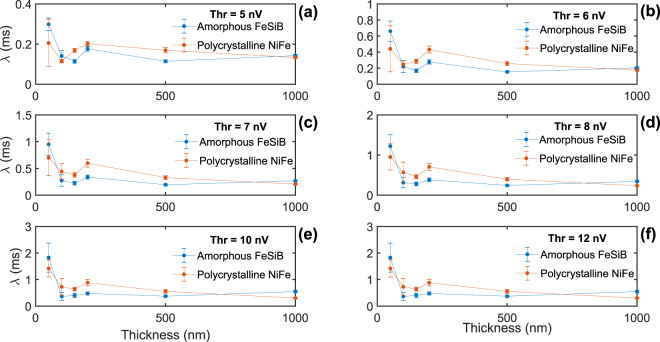
Dependence of the waiting-time statistics with film thickness and structural character of the materials. Typical inter-avalanche time *λ*, estimated from the exponential-like distributions of waiting times measured imposing distinct threshold levels to Barkhausen noise time series recorded in amorphous FeSiB and polycrystalline NiFe ferromagnetic films with different thicknesses. The error bars were estimated using the standard deviation.

Figure 4 depicts the general behavior of the typical inter-avalanche time *λ* with film thickness and structural character of the materials for selected threshold levels.

Strikingly, amorphous FeSiB and polycrystalline NiFe films share the same profile of *λ*, with roughly similar characteristic times for the same threshold level and the very same dependence with thickness. Generally, despite it is affected by the threshold level, *λ* remains relatively stable over a broad range of thicknesses, from 1000 to 200 nm, shrinks with the approach of the thickness to 100 nm, and abruptly increases for the films with thickness below 100 nm. The reproducibility of this general behavior despite modifications in parameters of the analysis evidences it is not an artifact of the threshold level or background noise present in the experimental recordings. As a consequence, we understand that the waiting-time statistics brings genuine information on the intrinsic underlying DW dynamics taking place in ferromagnetic films with distinct thicknesses.

## Discussion

Our findings raise interesting issues on the waiting-time statistics in magnetic systems, i.e. the impacts of the threshold level, as well as of the film thickness and structural character of the materials on the waiting-time distribution of Barkhausen noise in ferromagnetic films.

For crackling noise in general, looking primarily at the temporal avalanche statistics, distribution of avalanche durations discloses a power-law scaling behavior, characterized by a scaling exponent that is independent of the threshold level for a reasonable range of values, as long as it is not too small or too large^6,9^. This is especially true for the Barkhausen avalanches in magnetic systems, for both bulk samples and thin films. For our films, the distributions of durations and their power-law features may be looked in detail at refs. ^8,9,24,25^, as well as a general framework of the traditional avalanche statistical properties and the universality classes of Barkhausen avalanches in films may be checked in ref. ^9^. Specifically, the power-law relationship in the distribution of avalanche durations is an unequivocal signature revealing the lack of a characteristic scale in the durations.

From another perspective, a key evaluation of crackling noise is the distribution of waiting times separating the avalanches. Remarkably, in a number of systems, as earthquakes^26^, acoustic^27,28^ and light^29^ emission from fractures, compression of wood samples^30^, and porous materials^31^, the distributions of waiting times between avalanches were found to be of a power-law type^26–31^. This latter power-law-distributed waiting time informs the presence of correlations in the avalanche triggering process^19,21,32^, although it has been argued that temporal avalanche correlations in crackling noise simply result from the thresholding process used to define the avalanches^19,21,33^. However, it is unlikely that any fingerprint found here might suggest similar behavior for the distribution of waiting times in magnetic systems.

It is interesting to notice that the exponential behavior found in the cumulative distribution function of waiting times $P(\tau \ge t)$ is kept even if the threshold level is modified, at least within the employed range of threshold values, from 0 to 20 nV. Actually, it is worth remarking that such exponential behavior still remains with increasing the threshold level, even for values much above 20 nV, not shown here, despite the statistical analysis becomes poor due to the significant reduction in the number of events. In particular, similar exponential behavior in the distributions of the time between avalanches has been previously observed in for instance urban soundscape time series^34^. Remarkably, here no evolution in the form of the waiting-time distribution is observed as the threshold level is varied. This well-known feature, i.e. the evolution in the form widely found in many crackling systems^19,21,33^, is still under investigation in our magnetic systems and the reasons for its lack remains open so far. Anyway, the robustness of the exponential distribution of waiting times, as well as the absence of any sort of change in the form in the distribution from the exponential one towards an apparent power-law behavior, might suggest the avalanches are triggered by uncorrelated processes, somehow related to a Poisson process^19,21,32^. In this respect, it exposes the non-existence of correlations between avalanches^19,21,32^.

Nevertheless, we verify that the typical inter-avalanche time *λ* in the Barkhausen noise is strongly affected by the threshold level imposed to define avalanches and waiting times. In other words, the thresholding process interferes with the determination of the Poisson behavior of the waiting times^23^. Noticeably, choosing only levels above the experimental background noise, we find a continuous raise of *λ* with the increase of the threshold. Curiously enough, this relationship between typical inter-avalanche time and threshold is not a surprise, but it is somewhat expected. Specifically, first, as the threshold level increases, the smallest avalanches are not captured anymore, and the time interval between successive recorded avalanches naturally becomes larger. Further, it is reasonable the breaking of some avalanches into a few pieces by thresholding, what promotes the emergence of temporally correlated subavalanches that are part of the same underlying avalanche^19^, as well as of artificially-manufactured waiting-time intervals. These features acting together are understood as the responsible for the dependence of *λ* with the threshold. However, it is worth pointing out here we interpret the latter has minor contribution to the whole process, given that any change in the form in the waiting-time distribution is found.

At last, we observe that the typical inter-avalanche time *λ* in the Barkhausen noise is also dependent on experimental characteristics of the films. Our results uncover that the waiting-time statistics are dependent on the film thickness, although they seem to be insensitive to the structural character of the materials. Noticeably, amorphous and polycrystalline films present roughly similar characteristic times and the very same dependence with thickness.

Recalling previous reports of our group, it is worth remarking we are analysing here the waiting-time statistics of Barkhausen noise in films that belong to different classes of materials. Specifically, we have recently shown how scaling exponents and average shape of the Barkhausen avalanches evolve with the structural character of the materials and film thickness^8,9,24,25,35–38^, informing these features of the samples play fundamental role on the signatures of the underlying domain wall dynamics. Through the quantitative comparison between experiment and theoretical predictions, we have demonstrated that the key to the understanding of the Barkhausen avalanche statistics in ferromagnetic films resides in the interplay between system dimensionality and range of interactions governing the DW dynamics^9^. Then, we have revealed amorphous and polycrystalline films with distinct thicknesses split into three well-defined universality classes^9^—The first class includes amorphous films thicker than 100 nm presenting three-dimensional magnetic behavior with short-range DW surface tension governing the DW dynamics; Polycrystalline films thicker than 100 nm belongs to the second class, having three-dimensional DW dynamics governed by long-range dipolar interactions; Polycrystalline and amorphous films thinner than 50 nm in turn fall into the third universality class, both with a two-dimensional DW dynamics dominated by strong long-range dipolar interactions. So, here we probed for the influence of the film thickness and structural character of the materials on the DW dynamics and investigated the waiting-time statistics for distinct universality classes in an experimentally controlled manner.

Within this playground, our results provide experimental evidences that the waiting-time statistics are influenced by the film dimensionality, although they are insensitive to the range of the interactions governing the DW dynamics. Remarkably, amorphous and polycrystals share the same waiting-time statistics. This feature at a first glance might mislead us, apparently placing them into a single universality class and leading us to think in an universal behavior, irrespective of the range of the interactions governing the DW dynamics. However, the most striking finding here resides in the evolution of the typical inter-avalanche time *λ* with thickness, which is directly related to system dimensionality. Focusing on the results for the threshold level chosen right above the background noise for instance, we find $\lambda \sim 0.15$ ms over the broad range of thicknesses from 1000 to 200 nm, a subtle shrinkage to $\lambda \sim 0.1$ ms with the approach of the thickness to 100 nm, and an abrupt increase to $\lambda \,\mathrm{ > 0.2}\,$ ms for the films with thickness below $100$ nm. Thus, we verify that $\lambda$ is markedly distinct in films with different dimensionality. Remarkably, we find a peculiar behavior of $\lambda$ at the thicknesses $\sim 100$ nm, indicating an imminent transition in the temporal characteristics of the noise at the border between three- and two-dimensional magnetic behaviors. Indeed, an interesting feature here is related to the modification of $\lambda$ as the film thickness is reduced from $\sim 100$ to $50$ nm. We understand the change found in the $\lambda$ value may be ascribed to a modification in the critical behavior of the system occurring within the thickness range, i.e. the system passes from one universality class to another^9^. From our framework of universality classes informed before, we interpret the evolution of the typical inter-avalanche time as a signature of an universal restructuring associated to the dimensional transition of the magnetic behavior occurring as the thickness is reduced, from a three-dimensional DW dynamics observed in thick films to a two-dimensional regime, commonly verified for films thinner than $100$ nm^9^.

What insights can be drawn from our work? Here we have investigated the waiting-time statistics in the Barkhausen noise measured in amorphous and polycrystalline films having different thicknesses. Although our results have been extracted from a crackling noise by the thresholding process, we have not identified any apparent power-law-distributed waiting times, a feature very often found in a number of systems. On the contrary, we have uncovered a robust exponential behavior in the distributions of waiting times in Barkhausen noise, irrespective of the threshold level and kind of probed magnetic sample. This provides quantitative and fundamental test beyond the traditional avalanche distributions and their power-law scaling exponents, undoubtedly revealing here a lack of correlations between avalanches in magnetic systems. Further, we have observed the thresholding process intereferes with determination of the typical inter-avalanche time. Despite it, we have noticed both amorphous and polycrytalline films share the same profile of $\lambda$, with roughly similar characteristic times for the same threshold level and the very same dependence with thickness. Specifically, we have verified a systematic evolution in the characteristic time of the exponential process with the film thickness, which is directly related to system dimensionality. Hence, besides informing on the temporal avalanche correlations, our results disclose the waiting-time statistics in magnetic systems also bring fingerprints of the universality class of Barkhausen avalanches. Specifically, we interpret the evolution of the typical inter-avalanche time as a signature of a dimensional crossover in the DW dynamics taking place within the thickness range between $100$ and $50$ nm for both, polycrystalline and amorphous films. Thereby, given this dependence with the universality class of the avalanche dynamics, our findings trigger interesting challenges to theorists and further experimental investigations in diverse systems exhibiting crackling noise.

## Methods

### Ferromagnetic films

We investigated Barkhausen noise in amorphous Fe_75_Si_15_B_10_ (FeSiB) and polycrystalline Ni_81_Fe_19_ (NiFe) ferromagnetic films with thicknesses from $50$ to $1000$ nm. The films were deposited by magnetron sputtering onto glass substrates, with dimensions $10$ mm × 4 mm, covered with a 2-nm-thick Ta buffer layer. The deposition process was carried out using the parameters previously described in ref. ^9^. During the deposition, the substrate moved at constant speed through the plasma to improve the film uniformity, and a constant magnetic field of 1 kOe was applied along the main axis of the substrate in order to induce magnetic anisotropy. Detailed information on structural and magnetic characterizations may be found in refs. ^8,9,24,25,36–39^.

### Barkhausen noise experiments

All the experiments in this study were performed at room temperature. We recorded Barkhausen noise time series using the traditional inductive technique in an open magnetic circuit, in which one detects time series of voltage pulses with a pickup coil wound around a ferromagnetic material submitted to a smooth, slow-varying external magnetic field. In our setup, sample and pickup coils were inserted in a long solenoid with compensation for the borders to ensure an homogeneous magnetic field on the sample. The sample was driven by a triangular magnetic field, applied along the main axis of the sample, with an amplitude of 300 Oe, value high enough to saturate it magnetically. Here we performed experiments with driving field frequency of $0.05$ Hz. Barkhausen noise was detected by a pickup coil (400 turns, 3.5 mm long and 4.5 mm wide) wound around the central part of the sample. The Barkhausen signal was then amplified and filtered using a 100-kHz 12-dB/octave low-pass preamplifier filter (SR $560$ Stanford Research Systems), and finally digitized by an analog-to-digital converter board (PCI-DAS $\mathrm{4020/12}$ Measurement Computing) with sampling rate of 4 × 10^6^ samples per second. Barkhausen noise measurements were performed under similar experimental conditions. The time series were acquired just around the central part of the hysteresis loop, near the coercive field, where the DW motion is the main magnetization mechanism and the noise achieves the condition of stationarity^6,7,40^. It is worth mentioning that at a preanalysis stage, we employed a Wiener deconvolution^8^, which optimally filters the background noise and removes distortions introduced by the response functions of the measurement apparatus in the original voltage pulses, thus providing reliable statistics despite the reduced intensity of the signal. In addition, the amplifier gain and number of turns of the pickup coil have been divided out of all voltage data. In particular, for each ferromagnetic film, the following analysis was obtained from 200 time series.

### Waiting-time statistics

A central issue in our work is to properly define the waiting times between avalanches in the Barkhausen noise. In Fig. 1, we represent a brief sketch of the employed methodology. For each experimental Barkhausen noise time series measured in ferromagnetic films, we imposed a finite threshold level that distinguish the beggining and the end of the avalanches, with their sizes and durations, and the waiting times. Specifically, excursions of the signal above the threshold are identified as avalanche events; excursions below the threshold in turn are also found, with corresponding time intervals between the avalanches referred to as the waiting times. This standard procedure in the field follows closely the technique reported in ref. ^34^.

We first characterized the investigated samples and showed why they belong to different universality classes. We identified the universality class of Barkhausen avalanches by measuring the distributions of Barkhausen avalanche sizes and avalanche durations, the average size as a function of the avalanche duration, power spectrum, and the average avalanche shape. These results may be looked in detail at refs. ^8,24,25^, as well as a general framework of the traditional avalanche statistical properties and the universality classes of Barkhausen avalanches in films may be checked in ref. ^9^.

Here we obtained the waiting-time statistics by imposing distinct threshold levels in order to verify the impacts of our choice on the statistical results. We can figure out that, if we raise the threshold level, less avalanches will be captured and, as a consequence, the average waiting time between successive avalanches will become larger. But how does it affects the waiting-time statistics?

In this work, we focused on the study of the probability statistical distribution of the waiting times $P(\tau )$. Despite the intense debate in literaure on the form of this distribution^19–23^, we found that this distribution follows an exponential law. Specifically, we considered the form $P(\tau )\propto {e}^{-\tau /\lambda }$ and estimated the parameter *λ*, which has dimension of time and also has a clear physical interpretation. In fact, in the context of this statistical distribution, *λ* is the characteristic time of the exponential process^41^.

Here, we performed the fit of the exponential curve according to the Method of Maximum Likelihood (MML)^42^. To estimate the parameters with de MML method, we worked with the cumulative distribution $P(\tau \ge t)$. In particular, the probability distribution of the waiting times $P(\tau )$ is the derivative of $P(\tau \ge t)$. The use of the cumulative distribution improves the curve fitting, because the fluctuations in the curve are weakened in the cumulative sum^43^. As the derivative of the exponential function is also an exponential, we are able to find the slope of the exponential either by using the distribution of probability or the corresponding cumulative distribution.

## References

[1] Sethna JP, Dahmen KA, Myers CR (2001). Crackling noise. Nature..

[2] Houle PA, Sethna JP (1996). Acoustic emission from crumpling paper. Phys. Rev. E..

[3] Gutenberg, B. & Richter, C. F. *Seismicity of the earth and associated phenomena* (Princeton University, Princeton, 1954).

[4] Zapperi S, Vespignani A, Stanley HE (1997). Plasticity and avalanche behaviour in microfracturing phenomena. Nature..

[5] Field S, Witt J, Nori F, Ling X (1995). Superconducting vortex avalanches. Phy. Rev. Lett..

[6] Durin, G. & Zapperi, S. *The Science of Hysteresis: Physical Modeling, Micromagnetics and Magnetization Dynamics, vol. II, ch. III* (*The Barkhausen Effect)* (Cambridge University Press, Amsterdam, 2006).

[7] Colaiori F (2008). Exactly solvable model of avalanches dynamics for Barkhausen crackling noise. Adv. Phys..

[8] Papanikolaou S (2011). Universality beyond power laws and the average avalanche shape. Nat. Phys..

[9] Bohn F (2018). Playing with universality classes of Barkhausen avalanches. Sci. Rep..

[10] Lux T, Marchesi M (1999). Scaling and criticality in a stochastic multi-agent model of a financial market. Nature..

[11] Adamic LA, Huberman BA (2000). Power-law distribution of the world wide web. Science..

[12] Sethna JP (1993). Hysteresis and hierarchies: Dynamics of disorder-driven first-order phase transformations. Phys. Rev. Lett..

[13] Galuzio P, Lopes S, dos Santos Lima G, Viana R, Benkadda M (2014). Evidence of determinism for intermittent convective transport in turbulence processes. Phys. A.

[14] Narayan & Fisher (1992). Critical behavior of sliding charge-density waves in 4- *ε* dimensions. Phys. Rev. B..

[15] Rice J, Ben-Zion Y (1996). Slip complexity in earthquake fault models. Proceedings of the National Academy of Sciences of the United States of America..

[16] Cowie PA, Vanneste C, Sornette D (1993). Statistical physics model for the spatiotemporal evolution of faults. J. Geophys..

[17] Chen K, Bak P, Obukhov SP (1991). Self-organized criticality in a crack-propagation model of earthquakes. Phys. Rev. A..

[18] Bak P, Tang C (1989). Earthquakes as a self-organized critical phenomenon. J. Geophys..

[19] Laurson L, Illa X, Alava MJ (2009). The effect of thresholding on temporal avalanche statistics. J. Stat. Mech.: Theory Exp..

[20] Koivisto J, Rosti J, Alava MJ (2007). Creep of a Fracture Line in Paper Peeling. Phy. Rev. Lett..

[21] Janićević, S., Laurson, L., Måløy, K. J., Santucci, S. & Alava, M. J. Interevent Correlations from Avalanches Hiding Below the Detection Threshold. *Phy. Rev. Lett.* **117**, 230601 (2016). 10.1103/PhysRevLett.117.230601 27982624

[22] Janićević, S., Jovković, D., Laurson, L. & Spasojević, D. Threshold-induced correlations in the Random Field Ising Model. *Sci. Rep.* **8**, 2571 (2018). 10.1038/s41598-018-20759-6 PMC5803239 29416055

[23] Lebyodkin MA, Shashkov IV, Lebedkina TA, Gornakov VS (2017). Experimental investigation of the effect of thresholding on temporal statistics of avalanches. Phy. Rev. E..

[24] Bohn F (2013). Universal properties of magnetization dynamics in polycrystalline ferromagnetic films. Phy. Rev. E..

[25] Bohn F (2014). Statistical properties of Barkhausen noise in amorphous ferromagnetic films. Phy. Rev. E.

[26] Ben-Zion Y (2008). Collective Behavior of Earthquakes and Faults. Rev. Geophys..

[27] Salminen LI, Tolvanen AI, Alava MJ (2002). Acoustic Emission from Paper Fracture. Phy. Rev. Lett..

[28] Stojanova M, Santucci S, Vanel L, Ramos O (2014). High Frequency Monitoring Reveals Aftershocks in Subcritical Crack Growth. Phy. Rev. Lett..

[29] Tantot A (2013). Sound and Light from Fractures in Scintillators. Phy. Rev. Lett..

[30] Mäkinen T, Miksic A, Ovaska M, Alava MJ (2015). Avalanches in Wood Compression. Phy. Rev. Lett..

[31] Baró J (2013). Statistical Similarity between the Compression of a Porous Material and Earthquakes. Phy. Rev. Lett..

[32] Sánchez R, Newman DE, Carreras BA (2002). Waiting-Time Statistics of Self-Organized-Criticality Systems. Phy. Rev. Lett..

[33] Font-Clos F, Pruessner G, Moloney NR, Deluca A (2015). The perils of thresholding. New J. Phys..

[34] de Sousa IP, dos Santos Lima GZ, Sousa-Lima R, Corso G (2019). Scale-free and characteristic time in urban soundscape. Phys. A.

[35] Santi L (2006). Effects of thickness on the statistical properties of the Barkhausen noise in amorphous films. Phys. B.

[36] dos Santos Lima GZ, Corrêa MA, Sommer RL, Bohn F (2012). Multifractality in domain wall dynamics of a ferromagnetic film. Phy. Rev. E..

[37] Lima GZdS (2017). Universal temporal characteristics and vanishing of multifractality in Barkhausen avalanches. Phy. Rev. E..

[38] Durin G (2016). Quantitative Scaling of Magnetic Avalanches. Phy. Rev. Lett..

[39] Silva EF (2017). Thickness dependence of the magnetic anisotropy and dynamic magnetic response of ferromagnetic NiFe films. J. Physics D: Appl. Phys..

[40] Durin G, Zapperi S (2006). The role of stationarity in magnetic crackling noise. J. Stat. Mech.: Theory Exp..

[41] Meyer, P. L. *Introductory Probability and Statistical Applications* (Addison-Wesley, 1965).

[42] Clauset A, Shalizi CR, Newman MEJ (2009). Power-law distributions in empirical data. SIAM Rev..

[43] Goldstein ML, Morris SA, Yen GG (2004). Problems with fitting to power-law distributions. Eur. Phys. J. B..

